# Duration-dependent effects of the *BDNF* Val66Met polymorphism on anodal tDCS induced motor cortex plasticity in older adults: a group and individual perspective

**DOI:** 10.3389/fnagi.2015.00107

**Published:** 2015-06-05

**Authors:** Rohan Puri, Mark R. Hinder, Hakuei Fujiyama, Rapson Gomez, Richard G. Carson, Jeffery J. Summers

**Affiliations:** ^1^Human Motor Control Laboratory, School of Medicine, Faculty of Health, University of Tasmania, HobartTAS, Australia; ^2^Movement Control and Neuroplasticity Research Group, Department of KinesiologyKU Leuven, Belgium; ^3^School of Health Sciences, Federation University Australia, BallaratVIC, Australia; ^4^Trinity College Institute of Neuroscience and School of Psychology, Trinity College DublinDublin, Ireland; ^5^School of Psychology, Queen’s University BelfastBelfast, UK; ^6^Research Institute for Sport and Exercise Sciences, Liverpool John Moores UniversityLiverpool, UK

**Keywords:** *BDNF*, Val66Met polymorphism, transcranial magnetic stimulation (TMS), transcranial direct current stimulation (tDCS), motor cortex, older adults, corticospinal excitability, plasticity

## Abstract

The brain derived neurotrophic factor (*BDNF*) Val66Met polymorphism and stimulation duration are thought to play an important role in modulating motor cortex plasticity induced by non-invasive brain stimulation (NBS). In the present study we sought to determine whether these factors interact or exert independent effects in older adults. Fifty-four healthy older adults (mean age = 66.85 years) underwent two counterbalanced sessions of 1.5 mA anodal transcranial direct current stimulation (atDCS), applied over left M1 for either 10 or 20 min. Single pulse transcranial magnetic stimulation (TMS) was used to assess corticospinal excitability (CSE) before and every 5 min for 30 min following atDCS. On a group level, there was an interaction between stimulation duration and *BDNF* genotype, with Met carriers (*n* = 13) showing greater post-intervention potentiation of CSE compared to Val66Val homozygotes homozygotes (*n* = 37) following 20 min (*p* = 0.002) but not 10 min (*p* = 0.219) of stimulation. Moreover, Met carriers, but not Val/Val homozygotes, exhibited larger responses to TMS (*p* = 0.046) after 20 min atDCS, than following 10 min atDCS. On an individual level, two-step cluster analysis revealed a considerable degree of inter-individual variability, with under half of the total sample (42%) showing the expected potentiation of CSE in response to atDCS across both sessions. Intra-individual variability in response to different durations of atDCS was also apparent, with one-third of the total sample (34%) exhibiting LTP-like effects in one session but LTD-like effects in the other session. Both the inter-individual (*p* = 0.027) and intra-individual (*p* = 0.04) variability was associated with *BDNF* genotype. In older adults, the *BDNF* Val66Met polymorphism along with stimulation duration appears to play a role in modulating tDCS-induced motor cortex plasticity. The results may have implications for the design of NBS protocols for healthy and diseased aged populations.

## Introduction

Non-invasive brain stimulation (NBS) techniques such as transcranial direct current stimulation (tDCS) and theta burst stimulation (TBS) have delivered promising results in older adults by inducing long-term potentiation (LTP) and long-term depression (LTD) like effects upon corticospinal excitability (CSE), accompanied in some instances by corresponding changes in behavior (Zimerman and Hummel, 2010). Hummel et al. (2010) reported significant improvements in older adults on the Jebsen–Taylor Hand function test following the administration of 20 min of anodal tDCS (atDCS), relative to sham, which outlasted the stimulation period by approximately 30 min. More recently, Zimerman et al. (2013) reported significant enhancement in complex motor skill acquisition after older adults received 20 min of atDCS, with the effects lasting for at least 24 h. However, a crucial issue and one that must be addressed if aspirations for clinically relevant interventions are to be realized, is the high intra- and inter-individual variability that has been reported in response to various NBS protocols (Hamada et al., 2013; Hinder et al., 2014; Wiethoff et al., 2014). In this regard, the majority of previous studies suggesting that there are behavioral benefits of atDCS (Zimerman et al., 2013) have focused primarily on the mean effects observed at the group level, with little discussion of the degree to which each individual within the cohort responds to the intervention. Some of the aforementioned variability may be explained by factors such as gender, time of day, habitual activity levels, and genotype (Ridding and Ziemann, 2010), which are known to influence the response to NBS. A greater understanding of the influence of these factors is likely to be critical in determining whether NBS has any therapeutic utility.

One particular genetic factor, brain derived neurotrophic factor (*BDNF*), plays an important role in maintaining neuronal structure and function in the human brain. It is a major modulator of *N*-methyl-D-aspartate (NMDA) receptor dependent synaptic plasticity with its mature form (mBDNF) found to play a role in LTP (Figurov et al., 1996) and its precursor peptide form (pro-BDNF) implicated in LTD (Woo et al., 2005). One common single nucleotide polymorphism lies on the pro region of the *BDNF* gene at codon 66, which results in a non-conservative amino acid substitution of valine (Val) to methionine (Met), causing reductions in activity dependent BDNF secretion by 18% in Val66Met heterozygote and 30% in Met66Met homozygote mice (Chen et al., 2006). Consequently, carriers of the Met allele appear to show structural deficits such as reduced hippocampal neuronal integrity along with functional deficits as indexed by poorer episodic memory in young adults (Egan et al., 2003). Similarly, older Met allele carriers exhibit slower perceptual speed (Ghisletta et al., 2014) and reduced performance on cognitive tests of delayed recall, processing speed, and general intelligence (Miyajima et al., 2008).

In an influential study, Cheeran et al. (2008) reported a significant modulation of motor cortex plasticity by Val66Met polymorphism with Met carriers showing reduced LTP-like and reduced LTD-like plasticity in response to intermittent (nominally CSE-enhancing) and continuous (nominally CSE-diminishing) TBS protocols, respectively, relative to Val/Val carriers. More recently, Lee et al. (2013) observed that Val/Val homozygotes, but not Met carriers, showed an increase in CSE after a combined motor training and intermittent TBS (iTBS) paradigm. However, in some instances, it appears that Met carriers exhibit enhanced facilitatory responses compared to Val/Val homozygotes in response to NBS. Specifically, in a study comparing responses to iTBS and atDCS, Met carriers showed greater response to atDCS than Val/Val homozygotes, whereas only Val/Val homozygotes showed facilitation in response to iTBS (Antal et al., 2010). Consistent with Antal’s findings, Teo et al. (2014) reported greater facilitation 30–90 min following atDCS in Met carriers relative to Val/Val homozygotes. More recently, Strube et al. (2015) reported trend level increases in CSE for healthy Met carriers relative to the Val/Val group following atDCS.

In older adults, however, there is a paucity of research concerning the impact of the *BDNF* Val66Met polymorphism on use- and NBS-induced plasticity. McHughen and Cramer (2013) found no association between *BDNF* genotype and motor behavior or motor cortex plasticity following a 30 min hand training program in healthy older adults (mean age = 73.2 years). In regards to NBS-induced plasticity, we recently reported that there was no significant influence of this *BDNF* polymorphism on CSE following 30 min of 1 mA atDCS in healthy older adults (mean age = 68.3 years) with a relatively small sample (six older Met carriers; Fujiyama et al., 2014). Nevertheless, as genetic factors were not a primary focus, the study was not powered with these in mind, and thus it remains possible that some effects were not detected. Therefore, not only is the role of the *BDNF* Val66Met polymorphism in mediating responses to NBS in older adults inconclusive on a group level but also little has been reported of the variability on an individual level. Additionally, several studies suggest that there is an age-dependent reduction in LTP-like (Fathi et al., 2010) and LTD-like (Freitas et al., 2011) plasticity following NBS protocols, as well as comparable reductions in use-dependent plasticity (Rogasch et al., 2009). Accordingly, extended epochs of tDCS stimulation – which appear to be more effective at inducing greater corticomotor excitability (Jaberzadeh et al., 2012) – may be required for older adults. With a view to investigating this possibility, in the present study we examined the effects of both the commonly employed 10 min epoch, and a 20 min epoch of atDCS in two separate sessions. In light of the different degree of tDCS response engendered by the *BDNF* Val66Met polymorphism, we hypothesized that the effects of stimulation duration would not be expressed equivalently in older Met carriers and Val/Val homozygotes.

## Materials and Methods

### Participants

Fifty-four healthy older adults, recruited from the community and local university, aged between 60 and 82 years (mean age = 66.85 years, SD = 5.40 years; 32 females) completed two experimental sessions, each of 2 h duration. The Mini-Mental State Examination (Dick et al., 1984) was used to screen participants for cognitive deficits, with all participants scoring within a normal range (score ≥ 26). All participants were screened, via a medical history questionnaire, for contra-indications to transcranial magnetic stimulation (TMS) and tDCS and were free of any known neurological or neuromuscular dysfunction. Participants filled in a cardio respiratory fitness (CRF) questionnaire to record habitual levels of physical activity (Jurca et al., 2005). All participants provided written informed consent prior to participation in the study, which was approved by the Tasmanian Human Research Ethics Committee Network and conducted in accordance with the Declaration of Helsinki.

### *BDNF* Genotyping

All participants provided written informed consent prior to saliva collection using the Oragene DNA OG-500 collection kit. *BDNF* gene region rs6265 was amplified using ARMS-PCR (Sheikh et al., 2010). Three amplicons – two allele specific amplicons, 253 bp (val) and 201 bp (met) along with the 401 bp amplicon (entire rs6265 region as an internal control) were distinguished using four primers, namely, P1 forward CCTACAGTTCCACCAGGTGAGAAGAGTG, P2 (reverse) TCATGGACATGTTTGCAGCATCTAGGTA, P3 (G allele specific) CTGGTCCTCATCCAACAGCTCTTCTATAAC, and P4 (A allele specific) ATCATTGGCTGACACTTTCGAACCCA. The ARMS-PCR reaction consisted of a total volume of 12 μL – 1x MyTaq^TM^ Red Mix (Bioline, USA), 1 μM of each of the four primers (P1, P2, P3, and P4) and 10 ng of genomic DNA. Thermocycling conditions were as follows – denaturation at 94°C for 3 min followed by 30 cycles at 95°C for 45 s, 65°C for 60 s, and 72°C for 60 s, finished off with a final extension at 72°C for 2 min. Hypermarker IV (5 μL) and ARMS-PCR products were loaded onto a 2% agarose gel with electrophoresis carried out at a constant 100 V for 45 min. Based on the banding patterns, samples were classified as Val/Val (253/253 bp), Val/Met (253/201 bp), and Met/Met (201/201 bp) with all of them having the rs6265 internal control (401 bp) band. Each sample was genotyped from at least two independent polymerase chain reactions to ensure fidelity and based on their genotype, participants were grouped into either (i) being homozygous for the Val allele (Val/Val) – ‘Val/Val homozygotes’ or (ii) being heterozygous or homozygous for the Met allele (Val/Met, Met/Met) – ‘Met carriers.’ To avoid examiner bias, authors were blinded toward participants’ genotype until all NBS sessions were completed.

### Experimental Procedure

**FIGURE 1:**
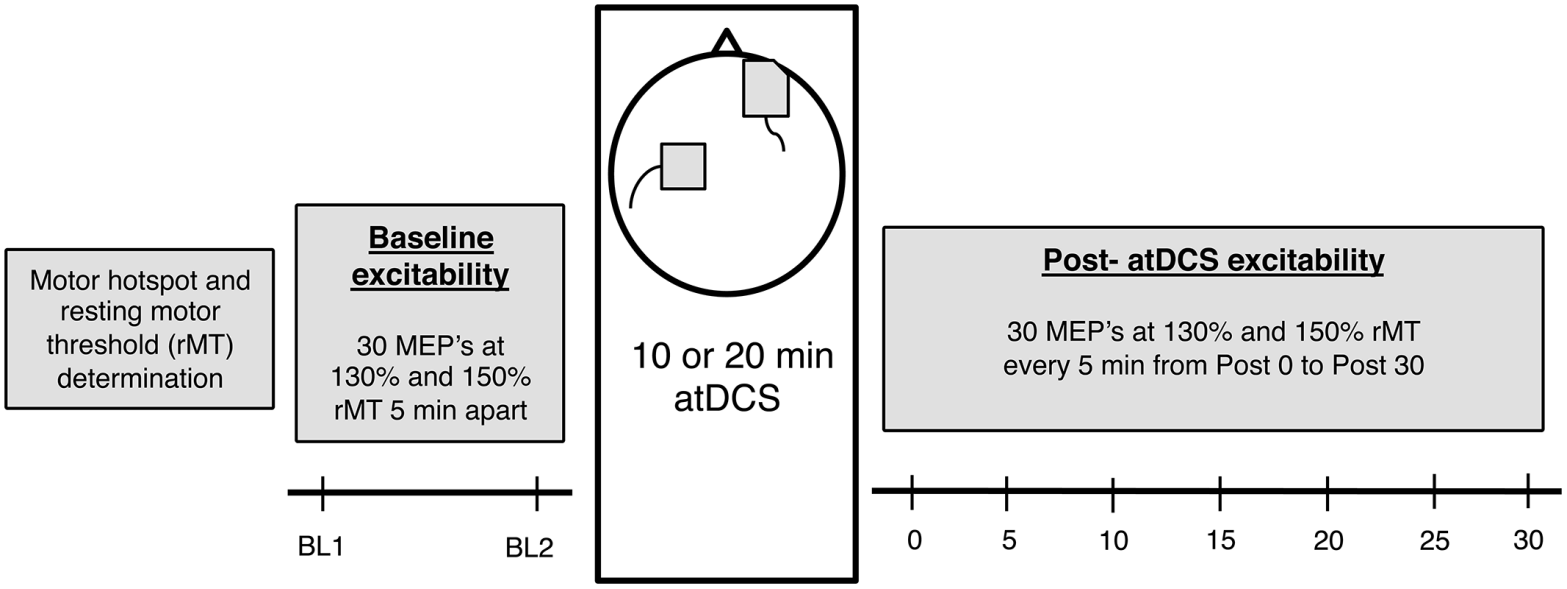
**The experimental design.** Firstly, for each participant, motor hotspot and resting motor threshold (rMT) was determined, after which baseline corticospinal excitability (CSE) was measured (two blocks, 5 min apart). Following this, 10 or 20 min anodal transcranial direct current stimulation (atDCS) was administered depending on the session, after which post-atDCS excitability was measured (7 blocks – Post 0 to Post 30, 5 min apart). Each block consisted of 15 neuronavigated TMS pulses at 130 and 150% rMT each [total 30 motor evoked potentials (MEPs)].

Participants attended two sessions of 2 h duration each and received either 10 or 20 min of atDCS in a counterbalanced order. Sessions were held at least 72 h apart to prevent any carry over effects from the previous session. Furthermore, both sessions were conducted at a similar time of day to account for the diurnal changes in cortisol which can affect CSE (Sale et al., 2008). Participants were comfortably seated in a chair with the right arm rested on a pillow and the left arm rested on their lap to minimize any muscle activation in the forearm and hand muscles. Following motor hotspot and resting motor threshold (rMT) establishment, baseline CSE was assessed in two separate blocks of TMS stimulation conducted 5 min apart. Participants then received 10 or 20 min of atDCS after which changes in CSE were recorded every 5 min for 30 min (**Figure 1**).

### Transcranial Magnetic Stimulation and Electromyography

EMG surface electrodes (Ag/AgCl), arranged in a belly tendon montage, were placed over the right first dorsal interosseous (FDI) muscle. Signals were amplified with a gain of 1000, band-pass filtered (20–1000 Hz), sampled at 4000 Hz using a 16-bit AD system (CED Power1401 and CED 1902, Cambridge, UK) and stored for oﬄine analysis. Using visual feedback, participants’ online EMG activity was monitored by the experimenter to ensure muscle relaxation and when necessary participants were reminded to keep their hand quiescent. Single pulse TMS was applied over the left motor cortex using a standard figure of eight coil (internal diameter of each wing was 70 mm) connected to a Magstim 200^2^ stimulator (Magstim Company, Dyfed, UK). The TMS coil was held tangentially to the scalp with the handle pointing ∼45° backward to ensure current flow in the brain was in the posterior–anterior direction. The motor ‘hotspot,’ determined using standard procedures, was marked on the scalp with a felt-tip pen and also co-registered to a neuronavigation device (Visor & Xensor TMS Neuronavigation, eemagine Medical Imaging Solutions GmbH, Berlin, Germany) to ensure consistent coil placement within each experimental session. Following this, rMT, defined as the lowest stimulator intensity required to evoke motor evoked potentials (MEPs) of ≥50 μV in 3 out of 5 consecutive trials for the right FDI (Carroll et al., 2001; Hinder et al., 2010) was determined for each participant at the beginning of the session. CSE was assessed at all time points using 30 single TMS pulses and a fixed inter stimulus interval of 5 s, with the order of the two stimulation intensities, 130 and 150% of rMT, varied randomly.

### Transcranial Direct Current Stimulation

Direct current stimulation was driven by HDCStim^TM^, a battery-operated constant direct current stimulator (Newronika s.r.l., Milan, Italy). Current was delivered through anodal (5 cm × 5 cm) and cathodal (6 cm × 8.5 cm) conductive rubber electrodes placed in saline soaked sponges with conductive gel. The center of the anodal electrode was placed over the FDI representation of the left primary motor cortex as determined earlier in the experimental session. The cathode was placed over the contralateral supraorbital region. After initial measurements of baseline CSE, participants received either 10 or 20 min of 1.5 mA anodal stimulation. The order of presentation of these conditions was counterbalanced across participants. There was an initial ramp up period of 7 s whereby current was linearly increased from 0 to 1.5 mA and maintained at this level for the duration of the intervention. Participants were reminded that they might feel a mild itching sensation under the electrodes. The electrode impedance was monitored throughout the session and always maintained below 10 kΩ.

### Data Processing, Analysis, and Statistical Procedures

Following collection of all data, participants were assigned to one of two groups on the basis of their *BDNF* genotype: “Val/Val” being homozygous for the Val allele and “Met carriers” being heterozygous and homozygous for the Met allele. Independent student’s *t* tests were then conducted to assess any potential age and CRF differences between the groups. CRF (measured in Metabolic Equivalents – METs) was calculated using parameters of age, gender, BMI, resting heart rate and self-reported physical activity level as proposed by Jurca et al. (2005). Fisher’s exact test was also conducted to reveal any association between gender and *BDNF* genotype.

Corticospinal excitability for a single trial was defined as the peak-to-peak MEP amplitude in the right FDI in a time window 10–100 ms following TMS. Trials in which root mean square (RMS) EMG activity exceeded 0.025 mV in a 50 ms time window immediately prior to the TMS pulse were excluded from the analysis. Repeated measures ANOVA revealed a grand mean RMS EMG value of 9 μV and no significant differences in EMG activity were observed between baseline and post-stimulation time-points for both atDCS sessions and intensities (see Supplementary Material). Following this, average peak-to-peak MEP amplitudes (in mV) were determined across the 15 trials at each intensity (130 and 150% rMT) and time points (two baseline and seven post-atDCS time points). Baseline differences in CSE between the two *BDNF* groups were compared using a four way mixed design ANOVA with factors of intensity (130 and 150% rMT), baseline (block 1, block 2), session (10 min atDCS, 20 min atDCS), and *BDNF* genotype (Val/Val, Met Carriers). Significant main and interaction effects were followed up with pairwise comparisons.

For each atDCS session, average MEP amplitudes at each of the seven post stimulation time points were normalized to the average MEP amplitude across both baseline blocks. These normalized MEP values violated the assumption of normality as revealed by significant Kolmogorov–Smirnov tests and were subsequently corrected using natural log transformations. Following this, a four way mixed design ANOVA with factors of intensity (130 and 150% rMT), time point (Post 0, 5, 10, 15, 20, 25, 30), session (10 min atDCS, 20 min atDCS), and *BDNF* genotype (Val/Val, Met carriers) was conducted on natural log transformed MEP amplitude. Significant main effects and interactions were followed up with relevant ANOVAs and pairwise comparisons to explore post-stimulation differences between the two groups for both atDCS sessions. For ease of interpretation and visualization, all mean values and figures utilize untransformed data (i.e., – normalized MEP amplitude). Accordingly, values > 1 indicates facilitation (increased excitability) and values <1 indicates suppression (reduced excitability), relative to baseline.

Intra-subject variability in response to the two different durations of atDCS was assessed qualitatively by constructing an *x*–*y* scatterplot. For each participant, normalized MEP amplitude values averaged across all post-stimulation time points were plotted for each atDCS session. Furthermore, the Fisher–Freeman–Halton exact test (Freeman and Halton, 1951) was conducted to test for any association between *BDNF* genotype and intra-individual variation in response to the different durations of atDCS. Inter-individual variability in response to atDCS was assessed by conducting a two-step cluster analysis using normalized MEP amplitude from Post 0 to Post 30 time points. Finally, a Fisher’s exact test was conducted to reveal any association between cluster membership and *BDNF* genotype.

IBM SPSS Statistics 21 (Armonk, NY, USA) was used for all statistical procedures with the *a priori* level of two-tailed significance set at 0.05. Bonferroni corrections were used to adjust for multiple comparisons. Greenhouse–Geisser adjusted values are reported if the assumption of sphericity was violated as indicated by a significant Mauchly’s test of sphericity (𝜀 < 0.7). Partial eta squared ($\eta_{p}^{2}$) or Cohen’s *d* values are provided as a measure of effect size for ANOVA and *t*-tests, respectively, and used to assist in the interpretation of inferential statistics.

## Results

All data is presented as means with 95% confidence intervals around the mean. Data from four participants was excluded from all of the following analyses due to excessively noisy EMG data at multiple time points. Genotyping analysis from the remaining 50 participants revealed that 37 participants were homozygous for the Val allele and 13 were Met carriers; this included 12 heterozygous and 1 homozygous for the Met allele. The distribution of the genotypes in our sample was in Hardy–Weinberg equilibrium (*p* = 0.98).

### Demographics and CRF for *BDNF* Genotypes

Val/Val genotypes (66.78 ± 1.75 years) did not differ significantly from Met carriers (67.23 ± 2.99 years) in age (*p* = 0.80, *d* = 0.08) or CRF levels (Val/Val: 7.80 ± 0.67 METs; Met carriers: 7.74 ± 1.00 METs; *p* = 0.93, *d* = 0.03). Fisher’s exact test (*p* = 0.52) showed no significant association between gender and *BDNF* genotype.

### Baseline Cortical Excitability

Four way ANOVA revealed no significant main effect of *BDNF* genotype, *F*(1,48) = 0.037, *p* = 0.849, $\eta_{p}^{2}$ = 0.001. There was a main effect of intensity, *F*(1,48) = 53.606, *p* < 0.001, $\eta_{p}^{2}$ = 0.528, with 150% rMT (2.94 ± 0.59 mV) eliciting larger MEPs than 130% rMT (1.64 ± 0.32 mV). A significant main effect of block, *F*(1,48) = 6.423, *p* = 0.015, $\eta_{p}^{2}$ = 0.118, was also observed with block 2 (2.38 ± 0.46 mV) exhibiting somewhat larger MEP responses compared to block 1 (2.20 ± 0.43 mV). Higher order interactions involving *BDNF* genotype and other factors of intensity (*p* = 0.993, $\eta_{p}^{2}$ < 0.001), baseline (*p* = 0.640, $\eta_{p}^{2}$ = 0.005) and session (*p* = 0.111, $\eta_{p}^{2}$ = 0.052) did not reach the *a priori* level of significance. Since the factor of baseline did not interact significantly with other factors in the analysis, MEP amplitude across both blocks was averaged and used as a measure of baseline cortical excitability in subsequent analyses.

### atDCS Induced Changes in Corticospinal Excitability: A Group Perspective

**FIGURE 2:**
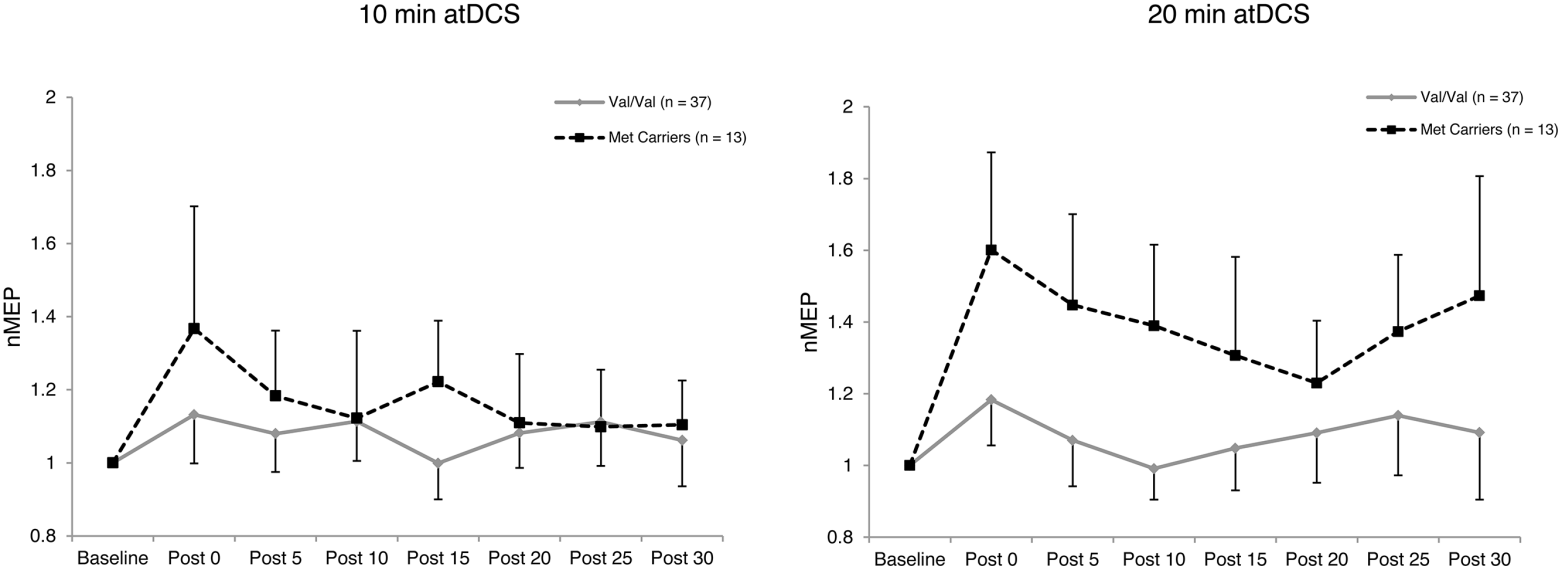
**Temporal pattern of post-stimulation response for each atDCS session and *BDNF* genotype.** Normalized MEP amplitude (ordinate) plotted for each atDCS duration (left panel – 10 min atDCS; right panel – 20 min atDCS) across all post stimulation time points (abscissa) for each brain derived neurotrophic factor (*BDNF*) genotype group (Val/Val group – solid line with diamond markers; Met carriers – dashed line with square markers). nMEP > 1 represents facilitation, nMEP < 1 indicates suppression. Error bars display the 95% confidence interval around the mean in one direction.

Four-way ANOVA on natural log transformed normalized MEP amplitude revealed a significant main effect of *BDNF* genotype, *F*(1,48) = 10.688, *p* = 0.002, $\eta_{p}^{2}$ = 0.182. Met carriers showed an enhanced response to atDCS (1.29 ± 0.11; 29% increase relative to baseline) compared to the Val/Val group (1.09 ± 0.06; 9% increase relative to baseline) as depicted in **Figure 2**. A significant four-way interaction, *F*(5.21,250.16) = 2.396, *p* = 0.036, $\eta_{p}^{2}$ = 0.048, was followed with two subsequent analyses.

**FIGURE 3:**
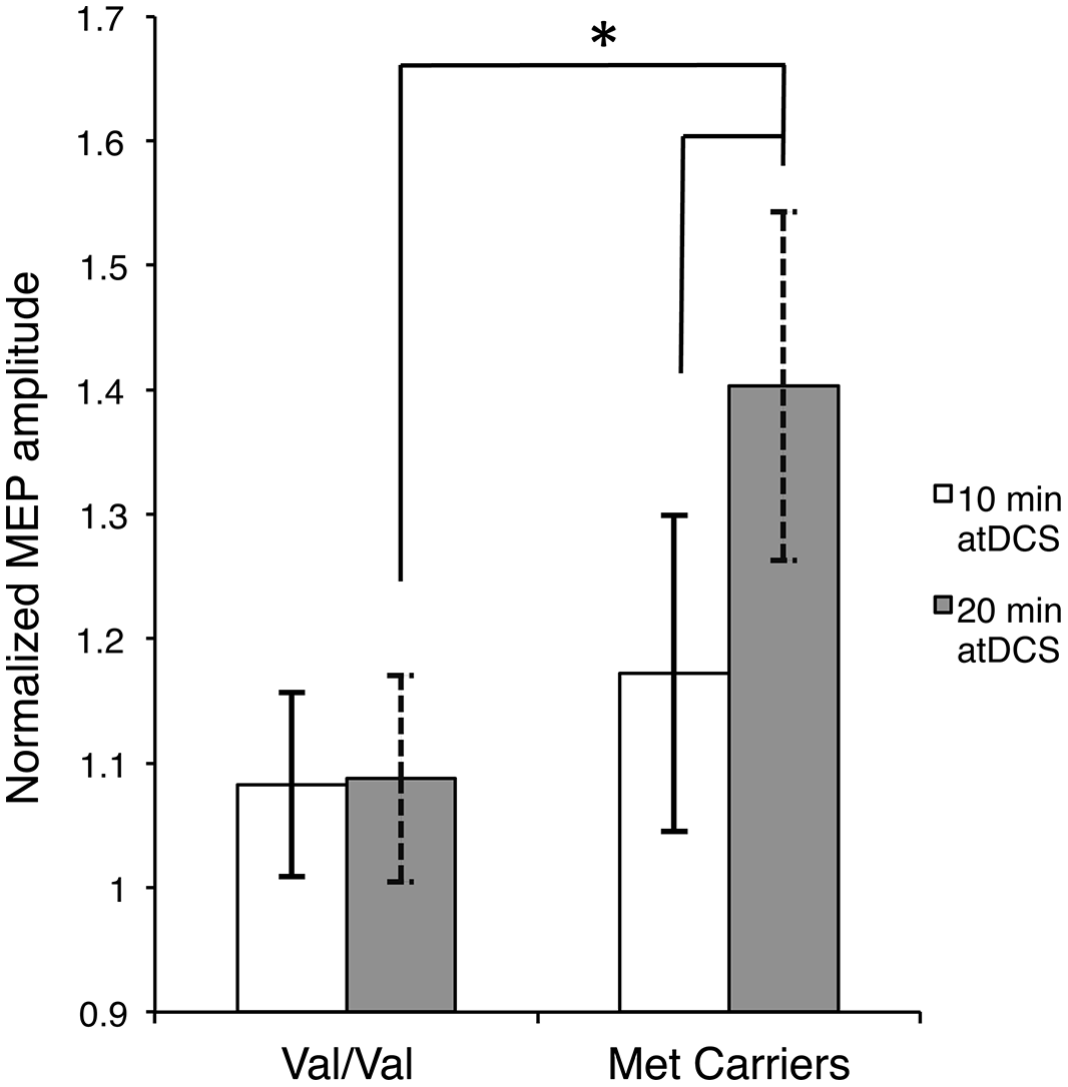
**Stimulation duration and *BDNF* genotype dependent differences in post-atDCS response.** Normalized MEP amplitude (ordinate), averaged over all post stimulation time points, in response to 10 min of atDCS (unfilled bars) and 20 min of atDCS (filled bars) plotted separately for each *BDNF* genotype (abscissa). nMEP > 1 represents facilitation, nMEP < 1 indicates suppression. Error bars display 95% confidence intervals around the mean and ^∗^*p* < 0.05.

Firstly, to reveal any differences between the groups in the temporal pattern of response to atDCS in the two sessions, we conducted separate two-way ANOVAs with the factors *BDNF* genotype and time point for each atDCS session. For 10 min of atDCS, no main or interaction effects were observed (*F’*s < 1.551, *p’*s > 0.219, $\eta_{p}^{2}$ < 0.031). However, for 20 min of atDCS, a main effect of *BDNF* genotype was observed, *F*(1,48) = 11.077, *p* = 0.002, $\eta_{p}^{2}$ = 0.188, with Met carriers (1.40 ± 0.18; 40% increase relative to baseline) showing a significantly greater change in excitability compared to the Val/Val group (1.09 ± 0.10; 9% increase relative to baseline) (**Figure 3**).

Secondly, we assessed differences within each *BDNF* group by conducting separate two-way ANOVAs with factors of time point and atDCS session for each *BDNF* genotype. No main or interaction effects reached significance (*F’*s < 1.645, *p’*s > 0.151, $\eta_{p}^{2}$ < 0.044) for the Val/Val group. Met carriers, on the other hand, showed a main effect of atDCS duration, *F*(1,12) = 4.955, *p* = 0.046, $\eta_{p}^{2}$ = 0.292, exhibiting significantly greater facilitation after 20 min (1.40 ± 0.18; 40% increase relative to baseline) of atDCS compared to 10 min (1.17 ± 0.13; 17% increase relative to baseline) as displayed in **Figure 3**. Other main or interaction effects did not reach the a priori level of significance (*F’*s < 1.202, *p’*s > 0.322, $\eta_{p}^{2}$ < 0.091).

### atDCS Induced Changes in Corticospinal Excitability: An Individual Perspective

**FIGURE 4:**
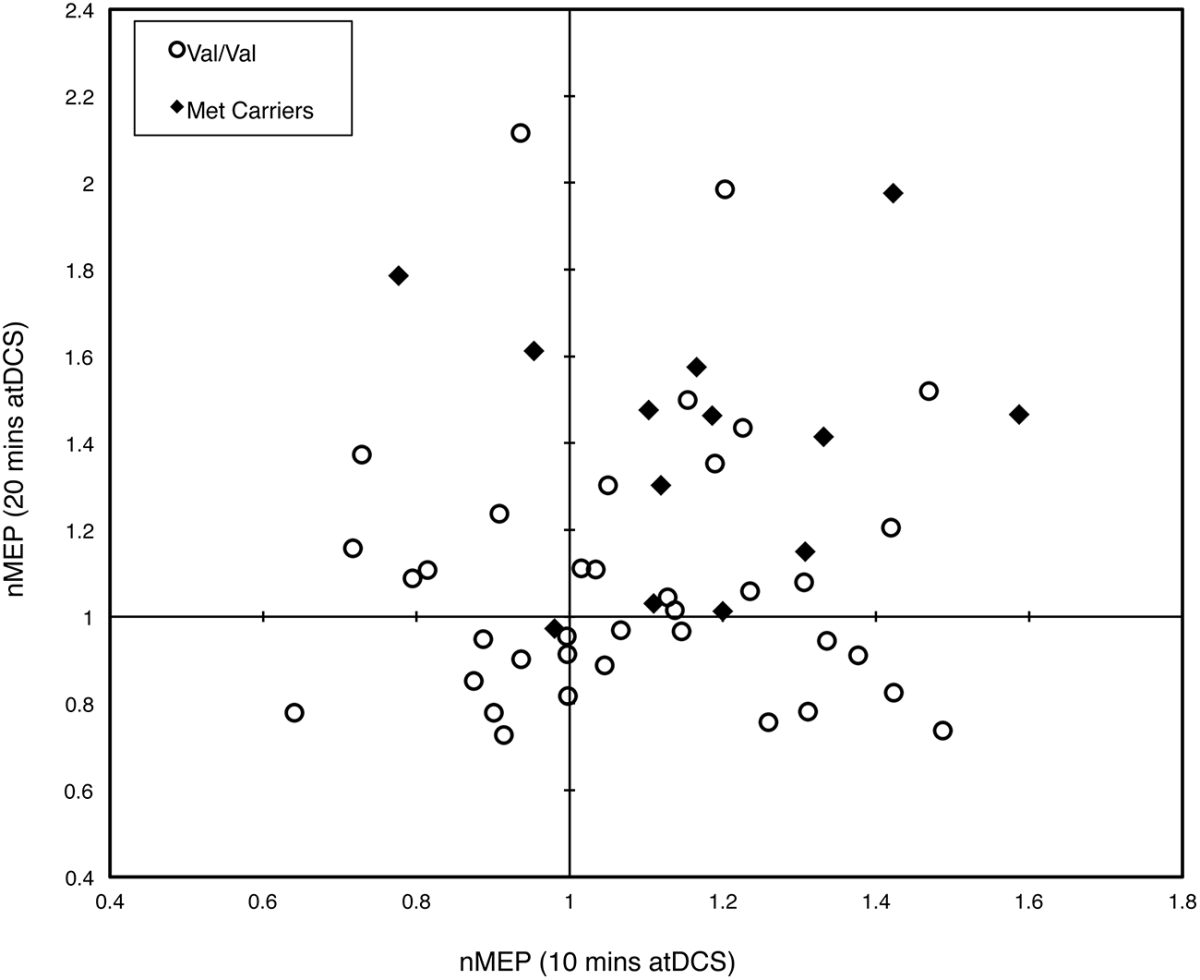
**Intra-subject variability in response to different durations of atDCS.** Scatter plot of normalized MEP amplitude averaged over all post-stimulation time points after 10 min of atDCS (abscissa) and after 20 min of atDCS (ordinate) for each participant. Participants are grouped by *BDNF* genotype, either Val/Val homozygotes (empty circles) or Met carriers (filled diamonds). nMEP > 1 represents facilitation, nMEP < 1 indicates suppression.

**Figure 4** depicts the individual post-stimulation responses to 10 min atDCS (abscissa) and 20 min atDCS (ordinate) using normalized MEP values, highlighting the variability in response to atDCS. Forty-six percent (46%) of participants (23 out of 50) showed the expected potentiation of MEP amplitude following atDCS in both sessions, while 20% of participants (10 out of 50) exhibited suppression of MEPs following atDCS in both sessions. Of the remaining 34%, 18% (9 out of 50) exhibited MEP facilitation following 10 min atDCS but MEP suppression following 20 min atDCS, while 16% (8 out of 50) showed the opposite effect, i.e., MEP suppression following 10 min atDCS and MEP facilitation following 20 min atDCS. The Fisher–Freeman–Halton exact test revealed a significant association (*p* = 0.040) between *BDNF* genotype and the characteristics of an individual’s response to atDCS (facilitation after both durations of atDCS, inhibition after both durations or facilitation after one and inhibition after the other). **Figure 4** illustrates that 77% of Met carriers (10 out of 13), but only 35% of Val/Val homozygotes (13 out of 37), showed the expected facilitation of MEPs in both sessions.

**FIGURE 5:**
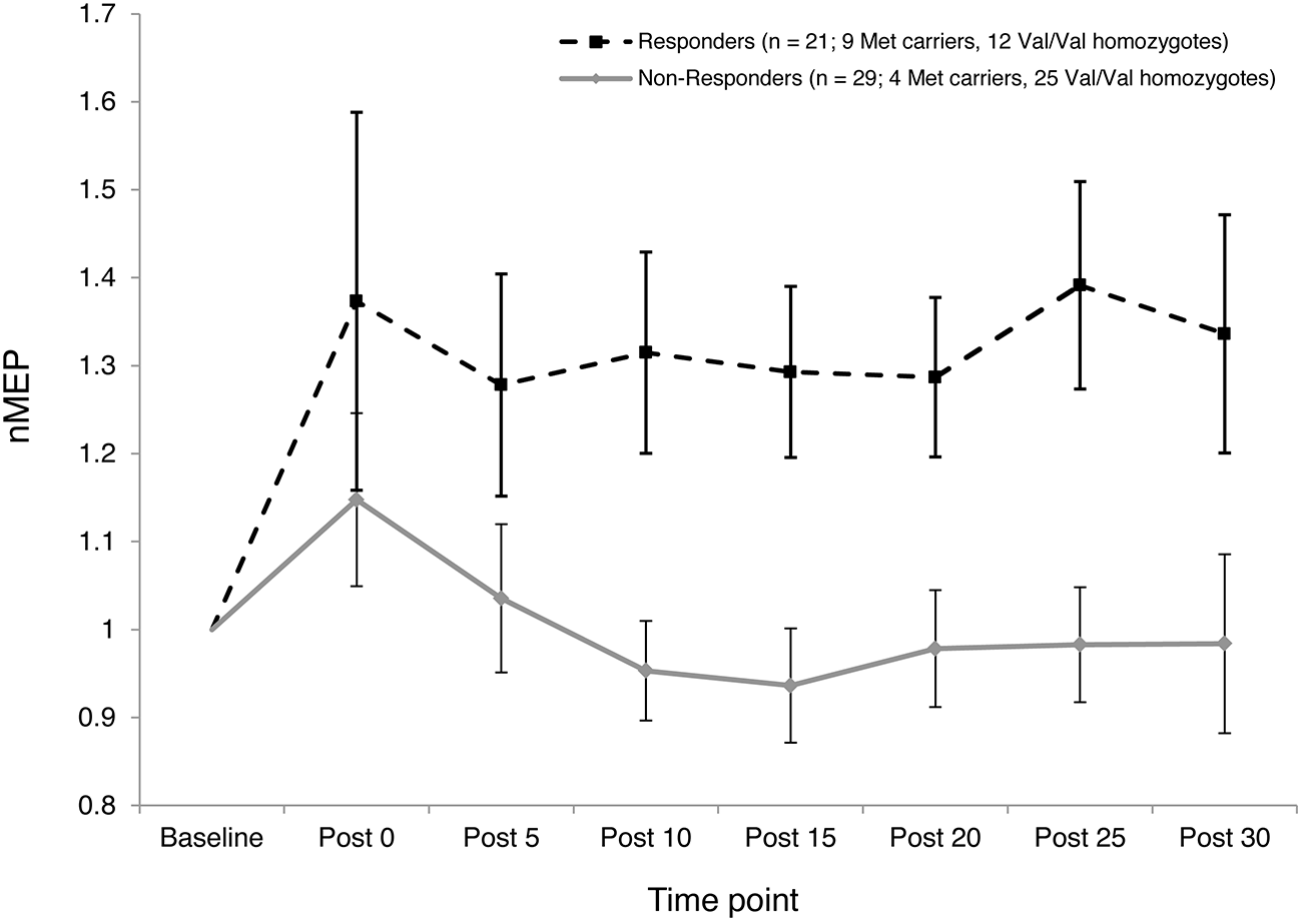
**Inter-subject variability in response to atDCS.** Normalized MEP amplitude (ordinate) averaged over both 10 and 20 min atDCS durations plotted at every post stimulation time point (abscissa) for each cluster (Responders – Dashed line with square markers; Non-responders – Solid line with diamond markers). nMEP > 1 represents facilitation, nMEP < 1 indicates suppression. Error bars at each time point display 95% confidence intervals around the mean.

The two-step cluster analysis revealed two distinct clusters of individuals with regard to their temporal responses to atDCS. Specifically, 42% of participants (21 out of 50) exhibited substantial post-stimulation MEP facilitation while 58% of participants (29 out of 50) exhibited little, or no, facilitation in response to atDCS or exhibited some MEP suppression (**Figure 5**). Fisher’s exact test revealed a significant association (*p* = 0.027) between *BDNF* genotype (Val/Val and Met Carriers) and cluster membership. Approximately two thirds, or 69%, of Met carriers (9 out of 13) belonged to the “responder” cluster, whereas only one third, or 32%, of Val/Val homozygotes (12 out of 37) were categorized as “responders.”

## Discussion

The current study provides new information concerning the role of the *BDNF* Val66Met polymorphism in moderating atDCS-induced motor cortex plasticity in older adults. Met carriers, but not Val/Val homozygotes, showed larger increases in CSE following 20 min of stimulation than following 10 min. Relatedly, the elevation of CSE induced by atDCS was larger for the Met carriers than for Val/Val homozygotes after 20 min but not after 10 min of stimulation. However, a substantial degree of inter- and intra-individual variability was observed. This is consistent with recent reports concerning tDCS (Lopez-Alonso et al., 2014; Wiethoff et al., 2014) and other NBS protocols, e.g., iTBS (Hamada et al., 2013; Hinder et al., 2014). The *BDNF* Val66Met polymorphism accounted for at least some of this variability. A greater proportion of Met carriers than Val/Val homozygotes showed facilitation to both durations of atDCS. In addition, a larger proportion of Met carriers than Val/Val homozygotes were classified as “responders” (**Figures 4** and **5**).

Three previous studies conducted in healthy young adults provide evidence that Met carriers exhibit greater increases in CSE in response to atDCS than Val/Val homozygotes. Specifically, Antal et al. (2010) reported that Met carriers (10 out of a sample of 24) exhibited greater facilitation following 7–9 min of 1 mA atDCS than Val/Val homozygotes. In a more recent study, whereby the numbers and demographics of the *BDNF* Val66Met polymorphism groups were matched, Teo et al. (2014) reported that Met carriers exhibited greater increases in CSE in the period 30–90 min following 9 min of 1 mA atDCS than Val/Val homozygotes. Similarly, Strube et al. (2015) reported trend level increases in CSE (*p* = 0.072; *d* = 0.799) following 13 min of 1 mA atDCS in Met carriers (8 out of a sample of 20) compared with Val/Val homozygotes. However, in older adults, the role of the *BDNF* Val66Met polymorphism is less well defined. We previously reported that *BDNF* Val66Met polymorphism did not play an evident role in mediating atDCS-induced M1 plasticity (30 min, 1 mA; Fujiyama et al., 2014). However, that study (19 participants; 6 Met carriers) was not powered with genotypic variations in mind. Based on the main effect of *BDNF* genotype, *F*(1,48) = 10.688, *p* = 0.002, the current study achieved a power of 89.3% (at 95% confidence) and an observed effect size of $\eta_{p}^{2}$ = 0.182. Based on this figure, a minimum of 38 participants would have been needed in the Fujiyama study to detect a significant effect attributable to *BDNF* genotype with 80% power. That study also employed lower tDCS intensity (1 mA) than in the present case. It has been suggested that higher tDCS intensities may yield greater post-stimulation neurophysiological (Nitsche and Paulus, 2001) and behavioral (Cuypers et al., 2013) change, and thus these may conceivably offer greater scope for modulation by other factors to be revealed.

Recently it has been suggested that prolonged durations of stimulation may lead to physiological responses that are of a sufficient magnitude to mask small variations otherwise attributable to genotype (Antal et al., 2014). In older adults – for whom, attenuated responses to various forms of brain stimulation have been reported (e.g., Fathi et al., 2010; Freitas et al., 2011), the threshold at which this masking occurs may well be different from that of younger adults. While our data are not consistent with this view, in so much as the largest differences attributable to genotype were obtained following 20 min of atDCS, there is as yet no basis upon which to conclude that this represents the asymptote of the response to this form of intervention. As such, further parametric variations of the stimulation protocol are likely to be required in order to provide a more complete exploration of this conjecture. In addition, our data shows preference of stimulation duration depending on the presence or absence of the *BDNF* Val66Met polymorphism, where only the Met carriers displayed significantly greater NBS-induced plasticity following 20 min compared to 10 min atDCS. Recently Hwang et al. (2014) reported greater cortical excitability in Val/Val homozygotes using suprathreshold high frequency repetitive transcranial magentic stimulation (rTMS) than was observed following subthreshold high frequency rTMS. In contrast, Met carriers did not exhibit this intensity-specific effect. Given that Met carriers appear to respond more readily to atDCS than Val/Val homozygotes [(Antal et al., 2010; Teo et al., 2014; Strube et al., 2015) and the current data] whereas the opposite appears to be the case following rTMS or iTBS [i.e., a propensity for Val/Val homozygotes to exhibit greater cortical change than Met carriers (Antal et al., 2010; Lee et al., 2013)], our current finding and Hwang’s could be seen to be complementary. Speculatively, these findings together suggest that the genotype that predisposes a greater response to a particular NBS (rTMS for Val/Val homozygotes; tDCS for Met carriers) may also prompt a greater response to higher stimulation intensity (rTMS) or longer stimulation duration (tDCS).

The classification of “responder” and “non-responder” groups on the basis of a two-step cluster analysis revealed a substantial degree of inter-subject variability in response to atDCS (**Figure 5**). As in previous studies that have employed a similar approach (Lopez-Alonso et al., 2014; Wiethoff et al., 2014), less than half of the current sample responded as expected (42% “responders”). This finding is also consistent with the extent of inter-individual variability reported in response to other NBS protocols such as paired associative stimulation (Muller-Dahlhaus et al., 2008) and iTBS (Hamada et al., 2013), whereby approximately half of the participants exhibit the anticipated response. Wiethoff et al. (2014), in trying to understand the causes of this inter-individual variability to atDCS, reported a moderate correlation between a surrogate measure of I-wave recruitment and response to atDCS, such that those who had a higher tendency to recruit early I-waves or D-waves showed the expected response to atDCS. While we did not record surrogate measures of I-wave recruitment, we found a significant association between *BDNF* genotype and cluster membership, whereby approximately two thirds of Met carriers were classified as “responders,” whereas only one third of Val/Val homozygotes fell in this category. Together, these findings indicate that there may be identifiable factors that account for inter-individual variability, and perhaps prognosticate responses to specific NBS protocols.

Intra-subject variability in response to varying durations of atDCS was also apparent. Approximately half (46%, 23 out of 50) of the participants exhibited some facilitation of CSE following atDCS in both sessions; a further one third of the participants (34%, 17 out of 50) exhibited a facilitatory response to atDCS in either the 10 or 20 min session, while the remaining participants (20%, 10 out of 50) exhibited some degree of corticospinal *suppression* following both sessions. However, given the design of the present study, it was not possible to distinguish intrinsic session-to-session variability (i.e., that which would be observed if the same duration of stimulation was repeated) from the variegated response that *may* have been attributable to manipulation of stimulation duration. Nonetheless, with recent research suggesting inter-session reliability of MEP responses for 30 min following atDCS (1 mA, 13 min) in 69% of young participants (Lopez-Alonso et al., 2015), the complex interplay between stimulation duration and *BDNF* genotype observed in the current study justifies further investigation.

One of the possible factors mediating the NBS-specific genetic modulation of plastic changes may be the mechanisms of action, both during and after stimulation, of the different NBS techniques. Anodal tDCS primarily affects resting membrane potential of neurons during stimulation with long-term effects mediated by changes in synaptic plasticity at glutamatergic and GABAergic neurons (Stagg and Nitsche, 2011). iTBS involves short trains of activity at the theta-frequency with after-effects thought to be at least in part NMDA-receptor dependent (Huang et al., 2007); however, recent evidence suggests that modulation of GABAergic neurons also occurs (Vidal-Pineiro et al., 2015). While not mutually exclusive with regard to the contributing mechanisms, subtle differences in the nature of the involvement of these mechanisms, and the interaction thereof with *BDNF* genotype, may explain the different influences of the Val66Met polymorphism on iTBS-induced and atDCS-induced gains in CSE. It has also been suggested that atDCS recruits D waves (Di Lazzaro et al., 2013) and early I waves (Lang et al., 2011), whereas iTBS-induced plasticity is thought to be primarily mediated via late I waves (Di Lazzaro et al., 2008), implying the possibility of different circuits being involved (Ziemann and Rothwell, 2000). Furthermore, differences in secretion of pro-BDNF molecules (significant reductions in Met carriers compared to Val/Val homozygotes; Chen et al., 2006) and pro-domain (cleaved from pro-BDNF) induced acute neuronal pruning (occurring only for Met carriers and not Val/Val homozygotes; Anastasia et al., 2013) attributable to the *BDNF* Val66Met polymorphism, are also likely to play an important role in modulating responses to different NBS techniques. It is crucial, therefore, that not only more systematic research be conducted in human subjects to elucidate these complex interactions but *in vivo* and *in vitro* animal studies also be conducted to understand the nature of interaction between BDNF and NBS protocols on a cellular and molecular level.

Among the limitations of the current study were that the number of Val/Val homozygotes and Met carriers were not matched. Notwithstanding the magnitude of the effects that were obtained, it is clearly the case that a larger sample size is to be preferred. It would have been an advantage to include a comparison group of young participants, and to utilize a sham stimulation condition. Moreover, the interaction of the *BDNF* Val66Met polymorphism with other polymorphisms (e.g., the *COMT* Val158Met polymorphism) is likely to play an important role as it has been shown that together these can modulate the response to NBS protocols such as paired associative stimulation (Witte et al., 2012). Recently, a proof-of-concept study reported an association of *BDNF* Val66Met polymorphism with cathodal tDCS induced plasticity in schizophrenia patients, warranting further research in clinical populations, especially those of the older demographic (Strube et al., 2015). In conclusion, our study highlights, at the group and individual level, that there may be an influence of the *BDNF* Val66Met polymorphism in mediating the changes in atDCS-induced motor cortex plasticity in older adults.

## Conflict of Interest Statement

The authors declare that the research was conducted in the absence of any commercial or financial relationships that could be construed as a potential conflict of interest.
