# Supplementary material for: Duration-dependent effects of the BDNF Val66Met polymorphism on anodal tDCS induced motor cortex plasticity in older adults: a group and individual perspective
Source: Front Aging Neurosci. 2015 Jun 5;7:107. doi: 10.3389/fnagi.2015.00107 (PMC4456583; doi:10.3389/fnagi.2015.00107)
Supplement: Supplementary file 1 [file Table_1.PDF]

Duration-dependent effects of the *BDNF* Val66Met polymorphism on anodal tDCS induced motor cortex plasticity in older adults: a group and individual perspective

Rohan Puri[1*], Mark R. Hinder[1], Hakuei Fujiyama[1,2], Rapson Gomez[3], Richard G. Carson[4,5], and Jeffery J. Summers[1,6]

1. Human Motor Control Laboratory, School of Medicine, Faculty of Health, University of Tasmania, Hobart, TAS, Australia

2. Movement Control and Neuroplasticity Research Group, Department of Kinesiology, KU Leuven, Belgium

3. School of Health Sciences, Federation University, Ballarat, VIC, Australia.

4. Trinity College Institute of Neuroscience and School of Psychology, Trinity College Dublin, Ireland

5. School of Psychology, Queen's University Belfast, Northern Ireland, UK

6. Research Institute for Sport and Exercise Sciences, Liverpool John Moores University, UK

* Correspondence:

Rohan Puri,
Human Motor Control Laboratory, Private Bag 30,
School of Medicine, University of Tasmania,
Hobart Tasmania 7001,
Australia
Rohan.Puri@utas.edu.au

Supplementary material

Repeated measures ANOVA of root mean square EMG values (in mV) was conducted with factors of intensity (130%, 150% rMT), session (10 min atDCS, 20 min atDCS) and time point (Baseline, Post 0, Post 5, Post 10, Post 15, Post 20, Post 25, Post 30) to ensure there was no significant differences in EMG activity at rest within a session and between sessions. IBM SPSS Statistics 21 (Armonk, NY, USA) was used with the *a-priori* level of two-tailed significance set at 0.05. Bonferroni corrections were used to adjust for multiple comparisons and Greenhouse-Geisser adjusted values are reported if the assumption of sphericity was violated as indicated by a significant Mauchly's test of sphericity ($\varepsilon < 0.7$). Partial eta squared (η_p^2) values are provided as a measure of effect size for the ANOVA and used to assist in the interpretation of inferential statistics.

The repeated measures ANOVA revealed a grand mean RMS EMG value of 9μV. No main effects of session, F (1, 49) = 0.168, p = 0.684, η_p^2= 0.003, intensity, F (1, 49) = 0.519, p = 0.475, η_p^2= 0.010 or time points, F (3.76, 184.20) = 2.461, p = 0.051, η_p^2= 0.048 were observed. Considering the effect size of the main effect of time points, post-hoc pairwise comparisons were carried out but no significant effects were revealed. No interaction effects reached statistical significance (Fs < 1.374, ps > 0.251, η_p^2 < 0.027). Descriptive statistics of RMS EMG values for all conditions are reported in Table 1 below.

Table 1 – Descriptive statistics (mean and standard deviation) of root mean square EMG values (in mV) for each time point (Baseline, Post 0, 5, 10, 15, 20, 25 and 30) and intensity (130% and 150% rMT) for both sessions (10 min and 20 min atDCS)

	Mean	Std. Deviation	N
Ten_130_Pre	.009124	.0016626	50
Ten_130_Post_0	.010103	.0038193	50
Ten_130_Post_5	.009233	.0016107	50
Ten_130_Post_10	.009066	.0012689	50
Ten_130_Post_15	.009202	.0018305	50
Ten_130_Post_20	.008915	.0009207	50
Ten_130_Post_25	.009210	.0015110	50
Ten_130_Post_30	.009207	.0017287	50
Ten_150_Pre	.009024	.0016603	50
Ten_150_Post_0	.009542	.0023603	50
Ten_150_Post_5	.009263	.0019810	50
Ten_150_Post_10	.009042	.0010943	50
Ten_150_Post_15	.009407	.0019758	50
Ten_150_Post_20	.008869	.0009150	50
Ten_150_Post_25	.009220	.0014537	50
Ten_150_Post_30	.009189	.0018144	50
Twenty_130_Pre	.008848	.0012979	50
Twenty_130_Post_0	.009379	.0024953	50
Twenty_130_Post_5	.009777	.0034294	50
Twenty_130_Post_10	.009159	.0018565	50
Twenty_130_Post_15	.008727	.0014303	50
Twenty_130_Post_20	.009153	.0017316	50
Twenty_130_Post_25	.009132	.0021579	50
Twenty_130_Post_30	.009019	.0021328	50
Twenty_150_Pre	.008718	.0011875	50
Twenty_150_Post_0	.009498	.0026651	50
Twenty_150_Post_5	.009583	.0036156	50
Twenty_150_Post_10	.009432	.0030503	50
Twenty_150_Post_15	.008826	.0013754	50
Twenty_150_Post_20	.008992	.0016629	50
Twenty_150_Post_25	.009066	.0015876	50
Twenty_150_Post_30	.009048	.0023185	50
